# Relation of leg phase angle from bioelectrical impedance analysis with voluntary and evoked contractile properties of the plantar flexors

**DOI:** 10.3389/fphys.2023.1292778

**Published:** 2023-11-23

**Authors:** Kosuke Hirata, Yosuke Yamada, Natsuki Iida, Akihiro Kanda, Mikio Shoji, Tsukasa Yoshida, Ryota Akagi

**Affiliations:** ^1^ Institute of Health and Sport Sciences, University of Tsukuba, Tsukuba, Japan; ^2^ Department of Physical Activity Research, National Institute of Health and Nutrition, National Institutes of Biomedical Innovation, Health and Nutrition, Settsu, Japan; ^3^ College of Systems Engineering and Science, Shibaura Institute of Technology, Saitama, Japan; ^4^ Mizuno Corporation, Osaka, Japan; ^5^ Graduate School of Engineering and Science, Shibaura Institute of Technology, Saitama, Japan

**Keywords:** bioelectrical impedance spectroscopy, triceps surae, muscle strength, rate of torque development, twitch contractile properties, electromyography

## Abstract

**Introduction:** Bioelectrical impedance analysis (BIA) can noninvasively and quickly assess electrical properties of the body, such as the phase angle. Phase angle is regarded as the quantity and/or quality of skeletal muscle and is associated with exercise performance, such as jump height and walking speed. Although the phase angle derived from BIA is assumed to be a useful way to assess muscle function, the relationship between the phase angle and neuromuscular properties has not been fully investigated. The purpose of this study was to investigate the association of phase angle with voluntary and evoked contractile properties in 60 adults (age, 21–83 years; 30 females and 30 males).

**Methods:** The phase angle of the right leg at 50 kHz was evaluated using BIA. The twitch contractile properties (peak twitch torque [PT_twitch_], rate of twitch torque development [RTD_twitch_], and time-to-PT_twitch_ [TPT_twitch_]) of the plantar flexors were measured using tibial nerve electrical stimulation. Maximal voluntary isometric contractions (MVICs) were performed to measure the maximal muscle strength and explosive muscle strength, from which the peak MVIC torque (PT_MVIC_) and rate of torque development (RTD) over a time interval of 0–200 ms were assessed, respectively. The root mean square (RMS) values of electromyographic (EMG) activity during the PT_MVIC_ and RTD measurements (EMG-RMS_MVIC_ and EMG-RMS_RTD_, respectively) were calculated. The RTD and EMG-RMS_RTD_ were normalized using PT_MVIC_ and EMG-RMS_MVIC_, respectively.

**Results and discussion:** Phase angle significantly correlated with twitch contractile properties (|*r*| ≥ 0.444, *p* < 0.001), PT_MVIC_ (*r* = 0.532, *p* < 0.001), and RTD (*r* = 0.514, *p* < 0.001), but not with normalized RTD (*r* = 0.242, *p* = 0.065) or normalized EMG-RMS_RTD_ (*r* = −0.055, *p* = 0.676). When comparing measurement variables between the low- and high-phase angle groups while controlling for sex and age effects, the high-phase angle group showed greater PT_twitch_, RTD_twitch_, PT_MVIC_, and RTD (*p* < 0.001) and shorter TPT_twitch_ (*p* < 0.001) but not normalized RTD (*p* = 0.184) or normalized EMG-RMS_RTD_ (*p* = 0.317). These results suggest that the leg phase angle can be an indicator of voluntary and evoked muscle contractile properties but not the neuromuscular activity of the plantar flexors, irrespective of sex and age.

## 1 Introduction

Bioelectrical impedance analysis (BIA) is widely used for estimating body composition. BIA noninvasively and quickly quantifies body electrical parameters (resistance and reactance) by sending a faint electric current within the body. Resistance is negatively associated with the water and electrolyte contents of the tissue, and reactance is positively related to the properties of the cell membrane capacitance (Barbosa-Silva et al., 2005). Phase angle is calculated as [arctangent (reactance/resistance) × 180°/π] (Baumgartner et al., 1988). Based on the characteristics of resistance and reactance and the equation of phase angle, a larger phase angle indicates a higher content of water-rich tissue within the body (e.g., muscle cell mass; Di Vincenzo et al., 2021) and better integrity of the cell membrane (Schwenk et al., 2000). The phase angle can be a proxy for muscle quantity/quality such as indices of sarcopenia (Di Vincenzo et al., 2021) and exercise performance, which is fundamentally influenced by skeletal muscle cell function. For instance, a larger phase angle is associated with a higher counter-movement jump height in young athletes (Bongiovanni et al., 2022) and faster 5-m walking speed in older adults (Yamada et al., 2019). Because the phase angle is non-invasively and quickly calculated by BIA without any muscle force production, it seems to be a convenient measure of exercise performance.

Exercise performance (such as jump height and walking speed) is influenced by several neuromuscular factors, including maximal muscle strength, the ability to generate rapid force, intrinsic contractile properties, and neuromuscular activity. Since the phase angle is well known to correlate with maximal muscle strength (Sacco et al., 2021; Akamatsu et al., 2022), one of the underlying mechanisms of the association between phase angle and exercise performance is the relationship between phase angle and maximal muscle strength. However, to the best of our knowledge, only one previous study has investigated the relationship of phase angle with rapid force generation ability, intrinsic contractile property, and neuromuscular activity at a single-joint level (Hirata et al., 2022b). This previous study reported that the phase angle derived from BIA correlated with electrically evoked twitch contractile properties (peak twitch torque [PT_twitch_], rate of twitch torque development [RTD_twitch_], and time-to-peak twitch torque [TPT_twitch_]) but not the rate of torque development (RTD; an index of rapid force generation ability) during maximal voluntary isometric contraction (MVIC) or electromyographic (EMG) activity in the knee extensors. Based on the results of this study, the phase angle may not correspond to rapid force generation ability or neuromuscular activity.

Although the phase angle is often measured at the whole-body level, particularly in studies on sarcopenia, it can also be measured at the segmental level. When estimating the function of a certain muscle group with the phase angle, it is better to measure the phase angle for a body segment that contains the target muscle group than for the whole body or other segments (Yamada et al., 2021; Hirata et al., 2022b; Bongiovanni et al., 2022). Because a body segment contains several muscle groups such as agonists, synergists, antagonists, phase angle measured for a certain body segment cannot assess function of only a target muscle group. The percentage volume of the knee extensors in the thigh is approximately 47%, whereas that of the plantar flexors in the leg is approximately 64% (Friederich and Brand, 1990; Ward et al., 2009). This implies that the assessment of muscle function using segmental phase angle is more detectable for plantar flexors than for knee extensors. Although a previous study (Hirata et al., 2022b) reported that the thigh phase angle did not correlate with rapid force generation or neuromuscular activity in knee extensors, these associations might be masked because of the relatively low percentage volume of knee extensors in the thigh compared with that of plantar flexors. Phase angle possibly relates to rapid force generation and neuromuscular activity because of the following reports: Yamada et al. (2017) reported that the phase angle evaluated from the lower extremities correlated with maximal power during a counter-movement jump, which is a multi-joint-level explosive strength. Second, the phase angle reflects the membrane integrity and function of the cell (Norman et al., 2012), and the properties of the cellular membrane are influential factors in neuromuscular activity (Farina et al., 2004). Furthermore, the membrane capacitance of the leg, which is closely related to the phase angle (Yamada et al., 2010), is associated with the EMG amplitude of the plantar flexors (Yamada et al., 2022). Therefore, exploring the relationship between leg phase angle and neuromuscular properties in the plantar flexors might be valuable for a better understanding of phase angle utility and the underlying mechanisms of the association between phase angle and exercise performance.

The purpose of this study was to investigate the association of the leg phase angle obtained by BIA with maximal muscle strength, rapid force generation ability, twitch contractile properties, and neuromuscular activity in plantar flexors. We hypothesized that leg phase angle would be associated with these variables. Specifically, there would be correlations of a larger phase angle with greater MVIC torque, RTD, PT_twitch_, RTD_twitch_, and EMG activity, and shorter TPT_twitch_.

## 2 Materials and methods

### 2.1 Participants

To calculate the sample size for single correlation analysis, G*Power statistical power analysis software (G*Power 3.1.7; Kiel University, Germany) was used to perform an *a priori* power analysis. The type 1 error and a statistical power were set at 0.05 and 0.80, respectively. According to a previous study (Akamatsu et al., 2022), we assumed an effect size of 0.40. The critical sample size was calculated as 44. We recruited 60 adults (21–83 years of age; 30 females and 30 males). The present study was part of a larger cross-sectional study conducted in our laboratory to investigate age-related differences in neuromuscular function (Hirata et al., 2022a; Yamada et al., 2022). The physical characteristics of the participants are summarized in Table 1. At the time of the experiment, none of the participants reported any muscle soreness, muscle fatigue, or orthopedic or neurological disorders. All participants were informed of the purpose and risks of this study and written informed consent was obtained from all participants. The ethics committee of Shibaura Institute of Technology approved the experimental procedure, and the study was performed in accordance with the Declaration of Helsinki.

**TABLE 1:** Anthropometric data, phase angle, twitch contractile properties, muscle strength, and neuromuscular activity of the participants. Bold values represent statistical significance.

	Pooled		Females		Males		*t*-test for sex	
	Mean	SD	Mean	SD	Mean	SD	*p* value	Cohen’s *d*
Anthropometric data								
Age (yr)	48	25	47	25	48	26	0.825	0.06
Height (cm)	163	9	156	6	169	6	**<0.001**	2.30
Weight (kg)	59.9	11.8	51.9	8.0	67.7	9.6	**<0.001**	1.82
Phase angle (degree)	6.2	1.0	5.8	0.9	6.5	1.0	**0.006**	0.74
Twitch contractile properties								
PT_twitch_ (Nm)	19.2	5.3	16.8	3.9	21.5	5.5	**<0.001**	0.98
RTD_twitch_ (Nm/s)	151	49	129	36	171	51	**<0.001**	0.99
TPT_twitch_ (s)	0.129	0.013	0.132	0.012	0.127	0.014	0.082	0.46
Muscle strength								
PT_MVIC_ (Nm)	100	34	81	26	118	30	**<0.001**	1.36
RTD (Nm/s)	283	130	224	108	341	125	**0.001**	0.93
nRTD (%MVC/s)	279	75	273	68	286	83	0.721	0.09
Neuromuscular activity								
nEMG-RMS_RTD_ (%MVIC)	79.6	21.5	87.7	21.8	71.7	18.4	**0.006**	0.75

SD, standard deviation; PT, peak torque; RTD, rate of torque development; TPT, time-to-peak torque; MVIC, maximal voluntary isometric contraction; nRTD: RTD, normalized by PT_MVIC_; nEMG-RMS_RTD_: root mean square value of electromyographic activity during RTD, normalized by that during MVIC.

### 2.2 Experimental procedures

The experimental procedures and settings conformed to those of our previous study (Hirata et al., 2022a). Briefly, the room temperature was set to approximately 23°C. Before BIA measurement, the participants were asked to lie supine on a stretching mat for 10 min to reduce the influence of body fluid shifts, caused by body postural changes, on the phase angle value. BIA measurements were performed three times in the supine position to assess the phase angle of the right leg. The participants were then asked to lie supine on a dynamometer bed (CON-TREX MJ; Physiomed, Germany) to evaluate the twitch contractile properties of the plantar flexors twice. Thereafter, muscle strength and neuromuscular activity of the triceps surae during MVIC were measured after several submaximal contractions as warm-up. The participants performed two types of MVIC tests to evaluate maximal muscle strength and explosive muscle strength. The maximal and explosive muscle strength trials were conducted twice and 10 times, respectively.

### 2.3 BIA measurement (phase angle)

The measurement details have been described previously (Yamada et al., 2010). Briefly, to ensure BIA data accuracy, the participants were asked to avoid eating, drinking, or bathing for 1 h and strenuous exercise for 24 h before the experiments. Participants lay supine on a stretching mat. Knee and ankle joints were relaxed, and hence these joints were approximately in anatomical position, i.e., thigh and shank longitudinal axes were almost parallel, and shank and foot sole were almost perpendicular. Electrodes for current injection (20 mm × 20 mm, Red Dot; 3M, United States) were attached to the dorsal surfaces of the right hand and foot. Sensing electrodes (20 mm × 20 mm, Red Dot; 3M, United States) were placed over the lateral aspect of the knee joint space between the lateral femoral condyle, lateral tibial condyle, and lateral malleolus of the right leg. The resistance and reactance of the right leg compartment were measured three times using an SFB7 (ImpediMed, Australia). Phase angle was measured using single frequency at 50 kHz, and calculated as arctangent of the ratio between the resistance and reactance [arctangent (reactance/resistance) × 180°/π] for each measurement. The mean values of the three phase angles were used for further analysis.

### 2.4 Twitch contractile properties measurement

The participants lay supine on the dynamometer bed and their hip, knee, and ankle joints were placed in an anatomical position. Their feet were fixed to the footplate of the dynamometer using a non-elastic strap. The rotational axes of the dynamometer footplate and ankle joint were visually aligned. The participants’ postures were the same throughout the following measurements: twitch contractile properties, maximal muscle strength, and explosive muscle strength. In order to electrically stimulate the tibial nerve, stimulation electrodes were attached over the popliteal fossa for cathode (20 mm × 20 mm, Red Dot; 3M, United States) and the frontal aspect of the thigh above the patella for anode (40 mm × 50 mm, Natus^®^ Disposable Adhesive Electrodes; Natus Manufacturing Limited, Ireland). Singlet electrical stimulation was conducted to elicit a twitch response from the plantar flexors using a constant-current variable voltage stimulator (DS7AH; Digitimer Ltd., United Kingdom). The stimulation intensity was set at 1.2 times the electrical current determined as the minimum intensity at which the twitch torque reached a plateau. PT_twitch_ was calculated as the difference between the baseline torque and maximal plantar flexion twitch torque. TPT_twitch_ was defined as the time interval from the onset of the twitch torque to the time point at which the maximal twitch torque was observed. RTD_twitch_ was computed by dividing PT_twitch_ by TPT_twitch_. The mean values of the two twitch responses were used for further analyses. The torque signal was stored on a personal computer through an A/D converter (PowerLab 16/35; ADInstruments, Sydney, Australia) using the LabChart software (ver.8; ADInstruments, Australia). The signal was digitized at 2 kHz and filtered using a 500 Hz low-pass filter.

### 2.5 EMG settings

A surface EMG system (Bagnoli 8 EMG System; Delsys Inc., United States) was used to evaluate the neuromuscular activity of the triceps surae. Skin preparation, shaving, abrasion, and cleaning with alcohol were performed. Subsequently, pre-amplified bipolar active surface EMG electrodes (electrode shape, parallel bar; electrode size, 1 mm × 10 mm; inter-electrode distance, 10 mm; DE-2.1, Delsys Inc., United States) were placed over each muscle belly of the triceps surae. The longitudinal locations of the electrodes were at proximal 30% of the leg length (distance between the lateral aspect of the knee joint space and the lateral malleolus) for the medial and lateral gastrocnemii, and midway between the distal myotendinous junctions of the lateral gastrocnemius and the distal myotendinous junctions of the soleus for the soleus. The transverse location of each electrode was at the center of the muscle width. The electrode was aligned with the fascicle direction of each muscle using an ultrasonographic apparatus (ACUSON S2000; Siemens Medical Solutions, Ann Arbor, MI, United States). A reference electrode was attached to the left lateral malleolus. The EMG signal filtered using 20–450 Hz bandpass filter by the EMG system was sampled at 2 kHz using LabChart software (ver. 8; ADInstruments, Sydney, Australia). The signal was synchronized with the torque signal.

### 2.6 Maximal muscle strength measurement

The participants exerted the MVIC torque twice for 4 s. They were provided with strong verbal encouragement. The peak torque of MVIC (PT_MVIC_) was calculated as the difference between baseline torque and highest torque value during plantar flexion MVIC. If the difference between the PT_MVIC_ values of the first and second trials was more than 10% of the highest value, a third trial was conducted. The maximum value of PT_MVIC_ among two or three trials was used for further analyses. Neuromuscular activity during MVIC for maximal muscle strength measurement was assessed using the EMG signals of the triceps surae. The RMS value of the EMG signal (EMG-RMS) was calculated over a 500-ms time window that included the time point of the highest torque value. The EMG-RMS value for the triceps surae during MVIC for maximal muscle strength measurement (EMG-RMS_MVIC_) was calculated by averaging EMG-RMSs for the medial- and lateral-gastrocnemii, and soleus. The EMG-RMS_MVIC_ was used for further analyses, because leg phase angle is the measure of entire leg muscle but not individual muscles.

### 2.7 Explosive muscle strength measurement

The participants performed 1-s brief MVICs for 10 times. The rest period between each brief MVICs was 20 s. They were instructed to exert force as hard and fast as possible and relax until just before a brief MVIC. Strong verbal encouragement was provided to each participant when performing the MVIC. If counter-movement (>0.3 Nm torque variation) or pre-activation (>3% of EMG-RMS_MVIC_) in the 200 ms prior to the onset of contraction was shown, or peak torque did not reach 70% of PT_MVIC_, such trials were excluded from the analyses. Onset was defined as the last trough before torque deflection above the baseline noise range (0.3 Nm) of the time-torque curve. The rate of torque development (RTD) was analyzed for the three brief MVICs containing the highest maximal instantaneous RTDs, which were calculated from the differential waveforms of the time-torque curves. The RTD was calculated as the slope of the time-torque curve over time intervals of 0–200 ms from the onset of contraction. EMG-RMS values during a brief MVIC for explosive muscle strength measurement were analyzed over the same RTD intervals (i.e., 0–200 ms) from the onset of EMG activity. EMG onset was manually identified as the last trough before EMG signal deflection above the range of the baseline noise (3% of EMG-RMS_MVIC_) of the rectified EMG signals. The EMG-RMSs obtained from the triceps surae were averaged and the mean value was defined as EMG-RMS_RTD_. The mean RTDs and EMG-RMS_RTD_ values calculated from the three brief MVICs that were selected were used for further analyses. To easily compare explosive muscle strength and neuromuscular activity during RTD among the participants, absolute RTD and EMG-RMS_RTD_ were normalized using PT_MVIC_ and EMG-RMS_MVIC_, respectively. These normalized values were named nRTD and nEMG-RMS_RTD_.

### 2.8 Statistical analyses

One female participant was excluded from all analyses because the number of successful RTD trials was fewer than three. As a result of the Shapiro–Wilk test, the normality of several parameters was violated. All data were statistically analyzed using log transformation. Sex difference in measurement variables was tested using independent t-test. A Pearson product-moment correlation analysis was conducted to test the association of the age with the phase angle and the association of the phase angle with the measurement variables of twitch contractile properties, muscle strength, and neuromuscular activity. To compare measurement variables between high- and low-phase angle groups while controlling for sex and age, one-way mixed-model analyses of covariance (ANCOVAs) were performed with the group (high-vs. low-phase angle group) as the between-subject factor (i.e., independent variable), measurement variables as the dependent variables, and sex [as a dummy variable (female = 0, male =1)] and age as covariate factors. Grouping was performed using 59 participants. The top 29 participants, in descending order of phase angle, were assigned to the high-phase angle group and the bottom 29 to the low-phase angle group. Significance level was set at *α* = 0.05, and effect sizes (Cohen’s d for *t*-test; r for main effects of one-way mixed-model ANCOVA; r for Pearson product-moment correlation analysis) were reported. All statistical analyses were performed using statistical software SPSS Statistics (version 28.0; IBM, Japan).

## 3 Results

### 3.1 Correlations of phase angle with measurement variables

**FIGURE 1:**
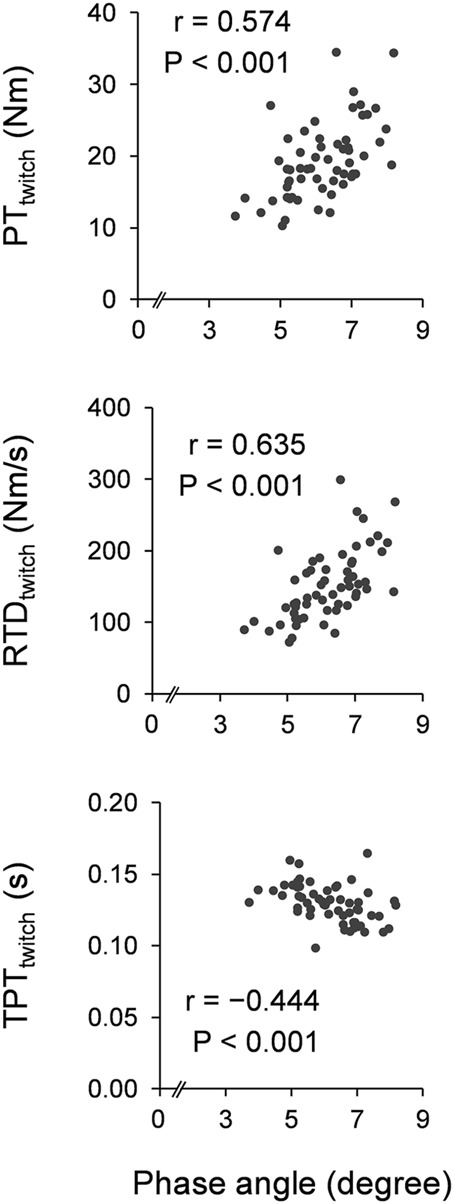
Scatter plots of phase angle with twitch contractile properties. Correlation coefficients and *p*-values were calculated by Pearson’s correlation analyses for log transformation values. PT, peak torque; RTD, rate of torque development; TPT, time-to-peak torque.

**FIGURE 2:**
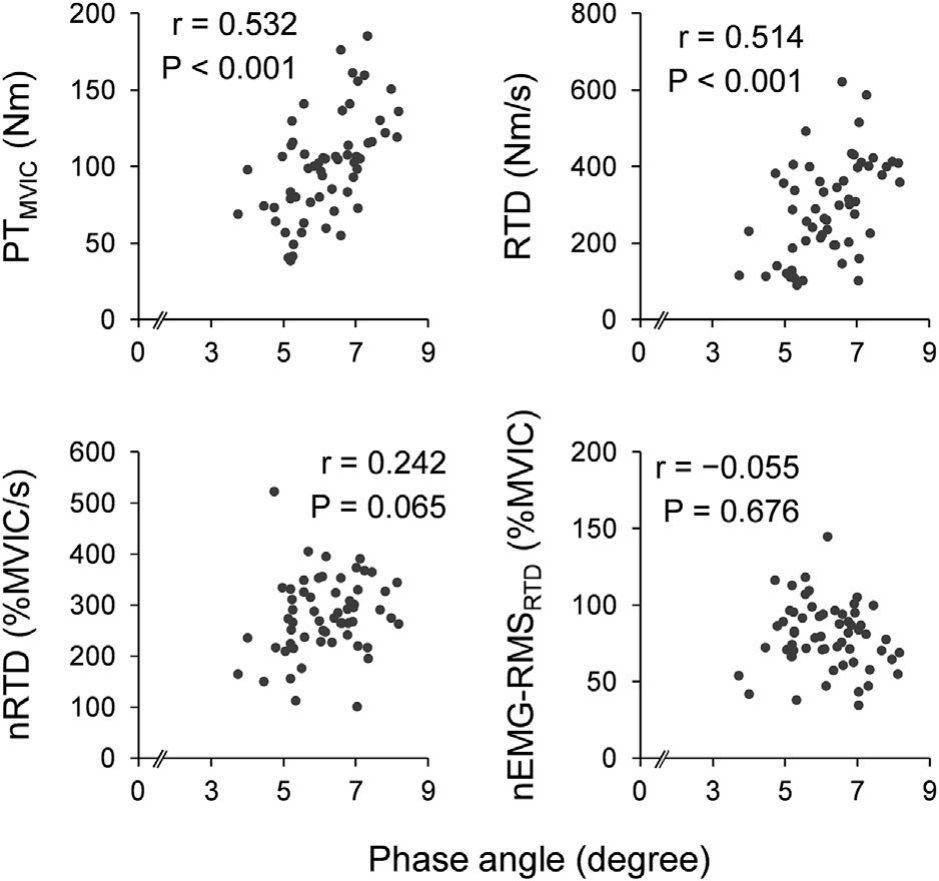
Scatter plots of phase angle with voluntary muscle strength and neuromuscular activity. Correlation coefficients and *p*-values were calculated by Pearson’s correlation analyses for log transformation values. PT, peak torque; MVIC, maximal voluntary isometric contraction; RTD, rate of torque development; nRTD: RTD normalized by PT_MVIC_; nEMG-RMS_RTD_: root mean square value of electromyographic activity during RTD normalized by that during MVIC.

Figure 1 shows scatter plots of the phase angle and twitch contractile properties. Leg phase angle significantly correlated positively with PT_twitch_ (*r* = 0.574 and *p* < 0.001) and RTD_twitch_ (*r* = 0.635 and *p* < 0.001), and negatively with TPT_twitch_ (*r* = −0.444 and *p* < 0.001). Figure 2 shows scatter plots of the phase angle with voluntary contractile properties (PT_MVIC_, RTD, and nRTD) and neuromuscular activity (nEMG-RMS_RTD_). Leg phase angle significantly correlated positively with PT_MVIC_ (*r* = 0.532 and *p* < 0.001) and RTD (*r* = 0.514 and *p* < 0.001), but not nRTD (*r* = 0.242 and *p* = 0.065) or nEMG-RMS_RTD_ (*r* = −0.055 and *p* = 0.676). Phase angle was negatively correlated with age (pooled: *r* = −0.590 and *p* < 0.001, females: *r* = −0.556 and *p* = 0.002, males: *r* = −0.738 and *p* < 0.001).

### 3.2 Difference in measurement variables between high- and low-phase angle groups

One-way mixed model ANCOVA (sex and age effects were controlled) revealed that the high-phase angle group had greater PT_twitch_ (*p* = 0.001 and *r* = 0.180), RTD_twitch_ (*p* < 0.001 and *r* = 0.249), PT_MVIC_ (*p* < 0.001 and *r* = 0.215), and RTD (*p* < 0.001 and *r* = 0.203), and shorter TPT_twitch_ (*p* = 0.001 and *r* = 0.168) than the low-phase angle group (Table 2). There were no significant differences in nRTD (*p* = 0.184 and *r* = 0.031) or nEMG-RMS_RTD_ (*p* = 0.317 and *r* = 0.018) between the groups.

**TABLE 2:** Differences in physical characteristics, phase angle, twitch contractile properties, muscle strength, and neuromuscular activity between the low- and high-phase angle (PhA) groups. Bold values represent statistical significance.

	Low-PhA group (*n* = 29) (17 females, 12 males)		High-PhA group (*n* = 29) (12 females, 17 males)		One-way mixed-model ANCOVA	
	Mean	SD	Mean	SD	P	r
Physical characteristics						
Age (yr)	63	19	34	21	—	—
Height (cm)	160	8	164	9	0.054	0.065
Weight (kg)	57.8	10.3	61.7	13.0	0.223	0.026
Phase angle (degree)	5.3	0.6	7.0	0.5	—	—
Twitch contractile properties						
PT_twitch_ (Nm)	17.0	4.2	21.4	5.5	**0.001**	0.180
RTD_twitch_ (Nm/s)	126	34	174	50	**<0.001**	0.249
TPT_twitch_ (s)	0.135	0.012	0.124	0.013	**0.001**	0.168
Muscle strength						
PT_MVIC_ (Nm)	84	27	116	33	**<0.001**	0.215
RTD (Nm/s)	230	114	338	126	**<0.001**	0.203
nRTD (%MVIC/s)	269	86	290	64	0.184	0.031
Neuromuscular activity						
nEMG-RMS_RTD_ (%MVIC)	82.7	20.3	77.6	22.3	0.317	0.018

ANCOVA, analysis of covariance; SD, standard deviation; PT, peak torque; RTD, rate of torque development; TPT, time-to-peak torque; MVIC, maximal voluntary isometric contraction; nRTD: RTD normalized by PT_MVIC_; nEMG-RMS_RTD_: root mean square value of electromyographic activity during RTD, normalized by that during MVIC.

## 4 Discussion

We aimed to clarify the association of phase angle obtained from the leg using BIA with voluntary muscle strength, twitch contractile properties, and neuromuscular activity. In line with our hypothesis, the correlation analyses elucidated the phase angle associated with twitch contractile properties (PT_twitch_, RTD_twitch_, TPT_twitch_), and absolute values of maximal muscle strength (PT_MVIC_) and explosive muscle strength (RTD). Contrary to the hypothesis, phase angle was not associated with the normalized values of explosive muscle strength (nRTD) and neuromuscular activity (nEMG-RMS_RTD_). These results were ascertained by comparing the high- and low-phase angle groups while controlling for sex and age effects. The present results suggest that individuals with larger phase angles can exert greater and faster muscle force and that the phase angle does not correspond to neuromuscular activity.

The leg phase angle was significantly associated with the twitch contractile properties of the plantar flexors (Figure 1). This supports the findings of a previous study (Hirata et al., 2022b), which elucidated the correlations between the thigh phase angle and twitch contractile properties of the knee extensors. Phase angle is a function of inverse of resistance. Since resistance is inversely associated with the water and electrolyte contents of the tissue (Barbosa-Silva et al., 2005), higher phase angle reflects higher water content. Especially in the limbs, most of the soft tissues are skeletal muscles which are the water-rich tissue. Hence, leg phase angle is considered as the leg muscle mass indicator. In addition, phase angle represents a balance between extracellular- and intracellular-fluid (Barbosa-Silva et al., 2005). Because extracellular matrix thickening was suggested to impair lateral force transmission (Zhang and Gao, 2014), larger phase angle which means less extracellular space leads to faster force transmission and greater force generation. Furthermore, phase angle associates with cell membrane integrity (Norman et al., 2012). Larger phase angle (fine cell membrane integrity) may be beneficial to transmit electrically evoked action potential on muscle cell membrane. These factors could be the underlying mechanisms of the association of phase angle with twitch contractile properties (PT_twitch_, RTD_twitch_, and TPT_twitch_). TPT_twitch_ is shorter in muscles with higher type II fiber content (Harridge et al., 1996). Aging decreases type II fiber content (Lexell, 1995), makes TPT_twitch_ longer (Phillips et al., 2022), and decreases the phase angle (Barbosa-Silva et al., 2005). This implies that the influence of age might derive a relationship between the phase angle and TPT_twitch_. However, a one-way ANCOVA with age as a covariate demonstrated that TPT_twitch_ was shorter in the higher phase angle group than in the lower phase angle group (Table 2). Therefore, phase angle could be associated with muscle fiber composition, although the reason for this remains unclear. It is possible that the phase angle is affected by fiber type-related differences in membrane electrical properties (Zengel et al., 1985), neuromuscular junction morphology (Sieck and Prakash, 1997), sarcoplasmic reticulum volume (Schiaffino and Reggiani, 2011), and aquaporin-4 expression in cellular membrane (Frigeri et al., 1998), because the phase angle is directly related to the amount and functional status of cell membranes (Barbosa-Silva et al., 2005).

The leg phase angle did not correlate with neuromuscular activity of the plantar flexors (i.e., nEMG-RMS_RTD_) (Figure 2). This supports the results of a previous study that investigated the knee extensors (Hirata et al., 2022b). Although the phase angle is suggested to reflect membrane integrity and function of cell (Norman et al., 2012), the membrane capacitance is a more direct bioelectrical impedance-derived index of properties of cell membrane. Skeletal muscle cells develop a tubular membrane system called the transverse tubule (T-tubule), which is essential to excitation-contraction coupling. T-tubule provides large membrane surface area of muscle leading to characterized larger membrane capacitance compared with other cells (Katz, 1948). Because the properties of the cellular membrane influence neuromuscular activity (Farina et al., 2004), larger membrane capacitance expects to relate to higher EMG activity. Indeed, the leg membrane capacitance evaluated by segmental bioelectrical impedance spectroscopy is reported to associate with the EMG amplitude of the plantar flexors (Yamada et al., 2022). Whereas the membrane capacitance is closely related to the phase angle (Yamada et al., 2010), these are not identical. Thus, although phase angle seems to have a potential to reflect neuromuscular activity, it may not be a good indicator like as membrane capacitance. Another reason for no correlation is that surface EMG activity reflects not only the peripheral properties but also the central properties of the neuromuscular system (Farina et al., 2004). In addition, when exerting explosive muscle strength, central nervous system (e.g., motor unit discharge frequency) plays an important role (Klass et al., 2008; Morel et al., 2015). Thus, peripheral aspects such as cellular membrane may not have a large impact on neuromuscular activity during explosive force generation.

The phase angle was significantly and positively correlated with voluntary muscle strength (i.e., PT_MVIC_ and RTD) (Figure 2). This result is in line with those of previous studies investigating the association of phase angle with the maximal strength of plantar flexors (Yoshida et al., 2018) and with PT_MVIC_ and RTD of the knee extensors (Hirata et al., 2022b). As mentioned earlier, phase angle associated with muscle quantity, muscle quality, and cell membrane integrity (Norman et al., 2012; Di Vincenzo et al., 2021), and significantly correlated with twitch contractile properties, which influence voluntary force generating capacity, in the current study. Therefore, the association between phase angle and voluntary muscle strength is reasonable. In contrast, the phase angle did not correlate with RTD normalized by PT_MVIC_ (i.e., nRTD) (Figure 2), whereas it correlated with RTD_twitch_ and TPT_twitch_. Normalized RTD is strongly affected not only by intrinsic contractile properties, but also by EMG activity (Folland et al., 2014). When performing RTD, the central nervous system is the main contributor, and the phase angle is not an indicator of central nervous system function. Indeed, there was strong positive correlation between nRTD and nEMG-RMS_RTD_ (*r* = 0.684, *p* < 0.001), and no correlation between phase angle and nEMG-RMS_RTD_ (*r* = −0.055, *p* = 0.676; Figure 2). Hence, no clear correlation between phase angle and nRTD was observed because of the influence of neuromuscular activity on nRTD in the present study. When we tried to test the association of phase angle with nRTD, controlling for EMG activity (nEMG-RMS_RTD_) by partial correlation analysis, a significant positive correlation was detected (*r*_*p*_ = 0.400, *p* = 0.002). Collectively, the phase angle can be an indicator of voluntary muscle contractile properties, whereas the phase angle is negligible but potentially associated with normalized explosive muscle strength.

Phase angle is the function of ratio of resistance (denominator) and reactance (numerator), and is indicators of muscle quantity, muscle quality, and cell membrane integrity (Norman et al., 2012; Di Vincenzo et al., 2021). These factors likely make connections of phase angle with voluntary and evoked muscle contractile properties. On the other hand, in this study, phase angle did not significantly associate with normalized rapid force generation ability (nRTD) and neuromuscular activity during RTD (nEMG-RMS_RTD_). This may be due to involvement in central nervous system. Several previous studies reported positive correlation between phase angle and exercise performance (Yamada et al., 2019; Bongiovanni et al., 2022). Exercise performance is affected by muscle strength, rapid force generation ability, and also motor control. Phase angle can estimate muscle contractile properties and hence exercise performance, while it should keep in mind that phase angle cannot reflect central nervous control.

The phase angle derived from BIA can be used to conveniently assess the physical condition of a multitude of people. Phase angle can be measured easily and quickly using BIA, without muscle contraction. Maximal muscle strength and rapid force production ability, which are associated with phase angle in the current study, are associated with sprint and agility performance (Wang et al., 2016), activities of daily living, such as rising from a chair and stair walking (Aagaard et al., 2010), balance control (Ema et al., 2017), and the risk of falls (Rubenstein, 2006). Therefore, the current results may help researchers, rehabilitation practitioners, and athletic trainers utilize phase angle for quick and secure assessments of muscle function and the physical condition of various individuals.

This study has some limitations. As mentioned in the introduction, BIA is a segment-level measurement. Hence, the phase angle measured by BIA was that of the entire leg muscles rather than just the plantar flexors. Nontarget muscle groups (e.g., dorsiflexors) can influence the association between phase angle and measurement variables. However, plantar flexors occupy approximately two-thirds of the leg muscle volume (Friederich and Brand, 1990; Ward et al., 2009). Therefore, the influence of nontarget muscles on the interpretation of the present results should be small.

## 5 Conclusion

The purpose of the present study was to investigate the association of leg phase angle obtained using BIA with maximal muscle strength, ability to generate rapid force, twitch contractile properties, and neuromuscular activity in the plantar flexors. A larger phase angle was positively correlated with greater maximal plantar flexion strength (PT_MVIC_) and explosive plantar flexion strength (RTD). Phase angle was also correlated with the twitch contractile properties (PT_twitch_, RTD_twitch_, and TPT_twitch_). These associations were confirmed after controlling for age and sex. Although the phase angle did not clearly correlate with nEMG-RMS_RTD_ or nRTD, a larger phase angle was associated with a greater nRTD when controlling for the influence of nEMG-RMS_RTD_. These results suggest that phase angle can be an indicator of voluntary and evoked muscle contractile properties but not of neuromuscular activity when developing rapid force. The association of phase angle with voluntary and evoked muscle contractile properties may be a potential mechanism for the connection between phase angle and exercise performance.

## Data Availability

The raw data supporting the conclusion of this article will be made available by the authors, without undue reservation.
